# Useful Methods of Diagnosis and Treatment

**Published:** 1908-08-22

**Authors:** John R. Keith


### The General Practitioner's Column.

[Contributions to this Column are invited, and if accepted will be paid for.]

### USEFUL METHODS OF DIAGNOSIS AND TREATMENT.

By JOHN R. KEITH, M.D.

There are a few methods of diagnosis and treatment which I have found very useful in practice, and which, as far as my experience goes, do not receive the attention they deserve. The first is

### Auscultatory Percussion.

Several years ago I came across a paper in the "International Clinics" which gave an exhaustive account of the subject, the perusal of which has enabled me to detect the true nature of various conditions which, without its help, I should have been quite unable to understand.

The method is best carried out by means of the old-fashioned wooden stethoscope, which is placed over an organ in the area in which it is in immediate contact, or almost immediate contact, with the body wall. Light percussion is made approaching or receding from the stethoscope, and it is then noticed that when made at one point the percussion reaches the ear forcibly, accompanied by a certain amount of shock or vibratory thrill, while a very short distance away (the fraction of an inch it may be) only a dull sound is heard, and no shock or vibratory thrill is noticed. In the former case, of course, percussion was made over the organ above which the instrument is placed, whilst in the latter its boundary has been passed.

By means of this method several obscure points may be determined with accuracy which otherwise would have to remain more or less problematical. For example, the lower margin of the left lobe of the liver may be defined even when intestine produces tympany on ordinary percussion. In many cases a contracted liver is liable to be diagnosed because the lower margin of a normal one has been obscured by resonant bowel which nullified the flatness of the liver tissue. Also malignant disease of the omentum may be quite undistinguishable from that of the liver. By auscultatory percussion, however, these conditions may be detected at once.

### Furunculosis Treated by Beer Yeast.

Although beer yeast has the approval of more than one distinguished dermatologist in the treatment of furunculi, in very few text-books is any allusion made to it. It will, however, often be found to succeed when the most vaunted drugs of the B.P. have been tried and found inefficacious. Fresh beer yeast is the best form of administration. The colour should be light chestnut. When allowed to settle in a glass it separates into three layers. At the bottom is a soft, thickish portion having the colour of dark coffee and milk; above that is a layer of dark chestnut-coloured liquid, while on the top by far the thickest layer is a sort of dense dark coffee-and-milk coloured cream that ferments with great activity. These three layers should be thoroughly mixed. The size of a dose varies from one to four teaspoonfuls. The smallest dose should be given at the beginning in case of gastric intolerance. It should be taken in a wineglassful of water (well stirred) three times daily at the beginning of each meal. It should be obtained fresh every day in summer and every second day in winter (when the weather is not particularly cold), and every third day if the weather is very cold. When beer yeast is not obtainable, baker's yeast may be tried instead. Of this a lump should be taken, the size of a hazel nut, mixed in a little water at each meal. Dr. Brocq, of Paris, the French dermatologist, declares that fresh beer yeast is as much of a specific in furunculosis as mercury in syphilis or quinine in malaria.

### Dislocations at the Hip-Joint.

A few months ago I was confronted with a thyroid dislocation at the hip-joint. I tried the usual method, but failed. Happily I had other help to appeal to. Nine years ago, in an odd quarter of an hour, I had copied from a medical publication into a notebook the details of a method of reducing hip-joint dislocations which the writer (Dr. C. A. Sturrack, of Dunfermline) stated he had found efficacious after the ordinary method had failed. I now found for the first time that my labour was well rewarded, for success was immediate and complete, the bone slipping into position with the utmost ease. The procedure is as follows: The patient, under anæsthesia, is laid on the floor in the supine position. The surgeon kneels upon his left knee when the left hip requires manipulation, and on the left side of the patient. The patient's thigh is then carefully flexed to a right angle, the leg being treated in the same manner, and laid with the most prominent part of the calf on the right knee of the surgeon. The ankle is then firmly grasped with the left hand and the condyles of the femur with the right. If it is a thyroid dislocation the thigh is abducted, and rotated in, by drawing the foot away from the middle line and keeping the knee steady. The ankle is then steadily depressed, the surgeon's knee acting as the fulcrum; and so powerful is the traction thus produced that the pelvis is easily raised from the ground. Then finally the thigh is rotated out, which is accomplished by keeping the knee steady and bringing the foot towards the middle line; and while this takes place the head of the femur returns to the acetabulum. Pubic and dorsal dislocations are treated in exactly the same manner except that the thigh is adducted instead of being abducted as in thyroid cases.

### Infantile Convulsions.

For long I treated these cases with bromides and chloral, given by the mouth or rectum, combined with hot blanket baths, etc. The results were tardy and often disappointing. Since, however, I have tried morphia hypodermically, success has been speedy and invariable. The dose required for an infant six months old is $\frac{1}{80}$ gr. (combined with atropine); for those of one year and two years, $\frac{1}{24}$ gr. and $\frac{1}{20}$ gr. respectively. In such doses there is no fear of the drug producing any dangerous narcosis.

